# Endorsing narratives of white replacement: Elite discourse, public attention, and extremist mobilization

**DOI:** 10.1371/journal.pone.0353353

**Published:** 2026-08-05

**Authors:** Jack Gabriel Risien Wippell, Laura Dugan

**Affiliations:** 1 Department of Sociology, The Ohio State University, Columbus, Ohio, United States of America; 2 The Mershon Center for International Security Studies, The Ohio State University, Columbus, Ohio, United States of America; Universidade Federal do Tocantins, BRAZIL

## Abstract

This paper examines how endorsements of white replacement conspiracies by political leaders might redirect public attention and energize white supremacist activity in the United States. We trace the historical evolution of replacement narratives and examine their appeal to political actors, then discuss theoretical frameworks linking reports of hostile political speech, public attention, and extremist mobilization. We construct a novel dataset combining federal and state legislators’ endorsements of replacement narratives that received media attention with Google search trends and indicators of white supremacist mobilization and propaganda. Using ARMAX time-series models, we identify a robust positive relationship between the cumulation of political endorsements and subsequent increases in white supremacist activity—a pattern that is replicated in state-level analyses employing state fixed-effects models. While cumulative endorsements are associated with public attention on immigration at the state level, this relationship is less pronounced at the national level, suggesting that legislators’ rhetorical influence may be spatially concentrated within their own constituencies. These findings reveal how political elites might simultaneously shift public attention and foster climates of outgroup hostility that increase extremist mobilization, offering broader insight into the interplay between public discourse about demographic change and socio-political behavior.

## Introduction

Demographic replacement conspiracies—that allege a deliberate effort to change a nation’s demographics and supplant a “true” people—are central to far-right narratives worldwide. In the United States, these beliefs center on claims that immigration is being strategically deployed to reduce White Christian conservatives to a minority [1,2]. For individuals experiencing increased anxiety amid economic instability and rapid social change, such conspiracies provide a comforting sense of certainty, offering clear targets for blame and straightforward solutions. While white nationalist groups have long propagated these conspiracies to attract members and fuel activism [3], part of a broader strategy of threat narratives [4,5], the rise of online forums and social media has amplified their reach [6]. In 2021, an AP-NORC poll indicated that nearly half of Republicans believe in a deliberate effort to “replace” native-born Americans with immigrants [7].

Extremist influencers often amplify conspiratorial ideas that are then circulated amongst everyday social media users. However, political elites also play a crucial role in shaping and disseminating these narratives for ideological or electoral gain [8]. Some leaders openly endorse extremist views, as seen in Donald Trump’s anti-immigrant “invasion” rhetoric and border policies, which fueled existing prejudices [9–11]. Indeed, a 2022 Yahoo/YouGov poll found that over 6 in 10 Trump supporters (61%) believed “a group in this country is trying to replace native-born Americans with immigrants and people of color who share their political views” [12]. Other times, however, politicians use subtler “dog-whistle” tactics, that resonate with mainstream audiences while “winking” to white supremacists, in what Sanchez describes as “rhetorical versatility” [13,14]. For instance, Trump’s “Make America Great Again” slogan is argued to evoke nostalgia for a past marked by heightened discrimination against racial, gender, and other minorities [15].

However, the influence of political narratives from elites likely extends beyond shaping public attention and opinion. Theory and empirical evidence suggest that political endorsements of hostile rhetoric may *embolden* those with existing prejudices to act [16]. Similarly, social movement scholarship on *discursive opportunities* [17], building from a long literature examining the cultural and political aspects of social movements [18–21], suggests these endorsements can be interpreted by movement adherents as signifying opportunities for mobilization. Both perspectives align with a substantial empirical literature linking anti-minority rhetoric and behaviors, including violence [22,23]. Yet, while these observed empirical links between political rhetoric and violence are compelling, we remain uncertain about how political endorsements of white replacement conspiracies, specifically, associate with extremist activity.

In this paper, we use a novel database that combines explicit public endorsements of white replacement conspiracies by federal and state legislators that were reported in major news outlets between 2021 and 2023, Google Trends data on attention towards immigration and white replacement conspiracies, and records of white supremacist protests and propaganda. We test hypotheses, at both national and state levels, that the cumulation of these political endorsements of white replacement conspiracies (1) directs public attention to immigration and replacement narratives and (2) fosters a climate of outgroup hostility that increases white supremacist mobilization.

We begin by situating this study within the broader history of white replacement conspiracies—particularly the prominence and diffusion of the “Great Replacement” narrative—to underscore both the persistence of these ideas across time and space and to clarify *why* political endorsements of such claims may be especially consequential. We then theorize several mechanisms through which elite endorsements shape (1) public attention and (2) mobilization among adherents, emphasizing the spatially bounded influence of politicians within their own constituencies, and the role of media coverage as the conduit through which these endorsements reach broad audiences. This leads to several hypotheses, which we test using weekly national time-series and also state fixed-effects models in the United States.

Our findings show general support for the hypotheses that the cumulation of political endorsements of white replacement conspiracies, reported by the media, is associated with increased white supremacist mobilization and public attention on immigration, though the latter relationship emerges most strongly at the subnational level. These results underscore the importance of sustained social scientific inquiry into the discursive dimensions of immigration’s growing politicization and the broader societal repercussions that may follow.

### A brief history of white replacement narratives

In 1903, *The Protocols of the Elders of Zion* was published in Imperial Russia (hereafter, *The Protocols*). The document purported to contain details of an international Jewish plot for global domination across 24 distinct protocols, which covered areas like banking, press censorship, religion, and education [24]. Despite eventually being exposed as a fabrication by *The Times* in 1921, *The Protocols* were treated as truth by many as they had already circulated widely across Europe, fueled by a climate ripe with antisemitism following far-reaching scandals such as the Dreyfus affair in France [25]. While not the only text to propagate these ideas, *The Protocols* was the most influential of its time and helped embed antisemitic conspiracy theories into the popular consciousness [26].

The tropes raised by *The Protocols* have periodically resurfaced across the West, most prominently through “white genocide” conspiracies [1,2]. Proponents of these conspiracies, drawing on resonant threat narratives [4,5], allege that Jewish “cabals” are orchestrating the erasure of the white race through mechanisms such as mass immigration, interracial marriage, and abortion [27]. In 1995, these ideas gained substantial traction in American politics when David Lane coined the “14 Words:” “We must secure the existence of our people and a future for white children.” This phrase continues to animate many white supremacist groups, including those who mobilize via otherwise “peaceful” protest and violence [28].

An important inflection point came in 2011 when Renaud Camus reformulated these ideas with the phrase “The Great Replacement” [29]. While often conflated with earlier versions of white genocide conspiracies, this iteration was particularly important in that it attributed agency for the “replacement” to a broader and decidedly ambiguous group of left-wing political leaders, while centering immigration as the primary mechanism of replacement [1]. Thus emerged a flexible and hostile framing–frames being the “schemata of interpretation” that aid individuals’ interpretation [30]–of immigration as an existential demographic threat, which hereafter we refer to as *white replacement conspiracies*.

Despite being repeatedly debunked, white replacement narratives have re-gained momentum within American politics. Influential individuals, including elected officials, have amplified these narratives to stoke “white anxieties” and sustain support for their agendas (Feola 2021). Indeed, Klofstad et al., (2025) found that 33% of Americans who were surveyed in 2022 believed in at least one narrative of white replacement [31]. In many ways, this shift accelerated under Donald Trump’s 2016 Presidential campaign, with repeated references to a Muslim “invasion” [32]. Equally notable was Trump’s response to the 2017 Unite the Right rally in Charlottesville–where participants chanted “Jews will not replace us,” describing it as “a display of hatred, bigotry, and violence” but with “good people on both sides” [33]. This response was seen by white nationalists as a legitimization of their conspiratorial beliefs.

These conspiracies were given another burst at life after the 2020 Presidential election with unfounded allegations of voter fraud. Political incumbents and candidates began invoking subtler replacement narratives, termed the “Great Replacement Lite” [34], that avoids explicit reference to racial replacement and instead claims left-wing politicians are orchestrating illegal immigration to increase Democratic votes. The long history of white replacement narratives, and their evolution toward ambiguous formulations centered on a malevolent “elite,” has rendered contemporary expressions of these ideas a form of “dog-whistle” politics [14]. Such statements are less likely to be perceived by the general public as overtly discriminatory, yet they remain legible to white supremacists and nationalists as endorsements of replacement conspiracies. As Benford and Snow note, frames are most powerful when they align with dominant public interpretations of the world [35,36]. The enduring cultural resonance of white replacement frames means political endorsements of these claims likely to be especially influential, even if explicit endorsements are comparatively rare.

During the 2024 Presidential campaign, these “Lite” conspiracies continued to feature within mainstream political discussion, such as when candidate Vivek Ramaswamy claimed that “Great Replacement is not some grand right-wing conspiracy, but a basic statement of the Democratic party’s platform” during the Republican primary debates [37]. Support for these narratives has also appeared across various levels of government. For example, in early 2024, Michigan State Representative Josh Schriver (R) shared a graphic on X that explicitly endorsed white replacement conspiracies. More recently, in May 2025, President Trump went so far as to present misinformation to South Africa’s President Ramaphosa, insisting that South Africa has been engaging in white genocide for years [38]. Yet, despite their increasing invocation by political elites, we currently lack a formalized understanding as to how these endorsements may shape socio-political life.

## Theoretical and empirical frameworks

How might elite endorsements of white replacement conspiracies—both their explicit (“main”) and coded (“lite”) forms—shape contemporary politics and society? In the sections that follow, we identify two key domains in which these endorsements are likely to exert influence. First, we argue that these endorsements are likely to direct public attention toward immigration. Second, we anticipate a mobilizing effect among white supremacist actors. We then consider the distinction between national and subnational influence for political leaders, and the ways that media coverage may condition the impact of these endorsements.

### Political discourse and public attention

Why do mainstream politicians and candidates continue to invoke these false claims about immigration? In political competition, public attention is a commodity to be won [39]. High-exposure messaging can be strategically used to shape political agendas, attract public support, and mobilize voters, helping politicians expand their influence [40,41]. With social media amplifying the speed and reach of information [42], this exposure can extend a politician’s profile well beyond their immediate base. However, with thousands of political figures vying for public attention, standing out from the crowd within this attention economy can be challenging. As Loseke [43] observes, “while many stories are told, only some circulate widely, and very few achieve widespread evaluations that they are believable and important.” This challenge is particularly acute for mainstream politicians, whose focus on complex policies often fails to captivate audiences.

One way that politicians can capture attention is through negative and emotive language, a well-documented strategy in research on political communication [44,45]. Studies show that negative posts, especially on social media, may be more engaging and widely shared than neutral or positive ones [46,47]. Emotional messaging can drive audience engagement [48], and studies suggest it is more potent when anger is directed toward an outgroup [49]. This dynamic is particularly relevant to the spread of conspiracies and hostile narratives, which often thrive during crises as individuals seek clear explanations and simple solutions [50]. Conspiracy theories, especially white replacement conspiracies, often carry intense negative emotions, framing minorities as existential threats. These narratives intertwine “symbolic codes” with “emotion codes,” casting specific groups as “villains” and evoking anger, condemnation, and a desire to punish [44]. Consistent with this perspective, Klofstad et al. (2025), using a 2022 U.S. national survey, find that anti-intellectualism and distrust in the scientific community are key predictors of belief in replacement narratives. This suggests that political elites may be able to mobilize support among these audiences by invoking such conspiracies [31]. In turn, conspiracy narratives may serve as a powerful tool for politicians seeking to elevate their visibility in a crowded political landscape. Note, of course, we recognize that explicit endorsements of racist beliefs continue to carry social stigma (Simi and Futrell 2009). However, such restraints do appear to be becoming less robust in the contemporary age. Belief in replacement conspiracies amongst Republican voters, as noted, has surged during the Trump era. As this social sanction is ameliorated, the costs of such endorsements relative to potential benefits decreases.

The political effectiveness of false, conspiratorial, and hostile rhetoric in shaping mainstream public attention was perhaps most evident in Donald Trump’s 2016 Presidential campaign. Despite more conventional candidates often leading in the polls, Trump was able to mobilize a mass following through populist, anti-immigrant messaging. Among the final four primary candidates–Trump, Cruz, Clinton, and Sanders–only Trump’s tweets and high-volume retweets consistently received substantial media coverage [51,52]. This strategy centered on deepening his base by fueling anger and encouraging activism rather than broadening his appeal to a wider electorate [53]. Thus, recognizing the capacity of political rhetoric to direct public attention, we expect such elite endorsements to associate with heightened public attention on the specific issues they invoke.

### Hostile rhetoric and hostile action

While online discourse is central to modern political life, the influence of political narratives extends beyond the digital sphere. Research increasingly suggests that political leaders can exacerbate societal divisions and incite violence through their messaging [16,54]. For instance, Feinberg et al. [55] found that counties hosting a 2016 Trump rally experienced significant increases in anti-immigrant hate incidents. This trend precedes the Trump era, as Dugan and Chenoweth [16] found that between 1992 and 2012, U.S. government actions against immigration were associated with a rise in violent hate crimes against Latinx individuals. This evidence aligns with a broader literature linking anti-minority rhetoric and mobilization [23]. As Hodwitz and Massingale [56] phrase it, Trump successfully fostered an “othering” dynamic, where the public is “primed to respond to leaders’ pejorative sentiments with thematically similar behaviors.”

The association between political hostility toward certain groups and mobilization against them is likely intensified when anti-minority rhetoric is couched within conspiracy beliefs. Research shows that such beliefs significantly elevate intentions toward violent extremism [57]. For example, belief in conspiracy theories linking 5G technology to COVID-19 has been associated with greater justification for violent action [58]. Similarly, evidence from a 2022 U.S. national survey indicates that individuals who endorse white replacement narratives are more willing to condone or use violence when they perceive the government to be acting against their values [31]. Violent extremist groups also routinely exploit conspiracy theories to legitimize and rationalize their violence [59]. With specific regard to white replacement narratives, it is notable that the perpetrators of several of the most high-profile lone-actor violent extremist incidents (e.g., the El Paso (2019), Buffalo (2022), and Jacksonville (2023) mass shootings) all referred to white replacement conspiracy theories in their manifestos [60].

Specifically, we theorize two main frameworks for understanding how elite endorsements may be linked to offline mobilization: *emboldenment* and *discursive opportunities*. Our focus is particularly on mobilization among those who hold white supremacist views, as such ideological alignment is a necessary precondition for mobilizing in response to endorsements of white replacement theories.

The first theoretical framework is *emboldenment* [16], which posits that statements of support by political elites can legitimize and thereby authorize individuals who hold these views to express them more openly. This framework extends classic sociological work on intergroup dynamics [61,62] by emphasizing how elite cues recalibrate perceived norms of acceptability. Although not always formally theorized in terms of emboldenment, similar logics appear across literature on elite rhetoric and violence against minority groups. Roscigno and colleagues [63], for example, show how elite framing of the Ghost Dance as a threat enabled violent repression against Native Americans, culminating in the Wounded Knee Massacre of 1890. Parallel dynamics have been identified at the individual level: Kalmoe [64] finds that exposure to violent political metaphors increases support for political violence among predisposed citizens. When elites publicly deploy hostile rhetoric toward specific groups, they signal that such expressions are now acceptable and, in doing so, empower adherents to act.

The second theoretical framework is one of *discursive opportunities* [17], which centers how shifts in public discourse, especially those initiated by political elites, may alter individual’s perceptions of the openness of the socio-political environment for mobilization. This scholarship has drawn insight especially from the intersection of political and cultural perspectives on movements [35]. For example, Molaei [65] finds that Indonesian social movements gained traction when online discourse converged with elite narratives. Central for our purposes is the notion of *consonance*, which Koopmans and Olzak [66] detail in a study on right-wing violence in Germany. When elite rhetoric tacitly or explicitly aligns with a movement targeting a minority group, it signals to adherents that their ideas are gaining traction within mainstream political discourse. This can lower the perceived risks of collective action, thereby encouraging mobilization to secure further gains that stem from visibility.

Taken together, these two frameworks illuminate complementary pathways through which elite discourse can animate collective behavior. *Emboldenment* emphasizes the psychological and normative effects of elite rhetoric, showing how legitimizing cues from authority figures can lower individuals’ expressive constraints and encourage action. In contrast, *discursive opportunities* focus on the structural and communicative dimensions of mobilization, showing how elite statements can signal that certain grievances now resonate with mainstream discourse and are politically viable. Drawing on both perspectives, we expect that public endorsements of white replacement conspiracies by political figures will spur activity among white supremacists, for whom these narratives most resonate.

### Additional considerations: media pathways and spatial concentration

Two additional considerations are essential for understanding how political leaders’ endorsements of replacement conspiracies may produce real-world consequences.

A first consideration relates to how elite rhetoric reaches and resonates with the public. Media systems play a central role in this process, serving as a prime conduit through which elite discourse is transmitted, interpreted, and amplified [40,41]. Coverage by mainstream outlets can substantially broaden the audience for political messaging, thereby increasing both public awareness and the perceived opportunity for action. Even critical or condemnatory coverage can lend visibility and a degree of legitimacy to a narrative by framing it as a matter of valid public debate rather than fringe belief [66]. This observation echoes a long-standing body of research emphasizing the media’s dual role as both amplifier and gatekeeper in shaping movement success and mobilization [67]. Indeed, it also resonates with foundational social movement framing literatures on how movements seek to amplify their interpretations of the world. In this light, attention to high-profile endorsements that have broken into the media cycle is especially important: elite statements that receive substantial media coverage are precisely those most likely to shape public attention and activate mobilization.

Second is the spatial scale of elite influence. Within the United States’ federal system, the authority of elected officials is often geographically bounded to (or at least concentrated within) the constituency they represent, making the state or local district a vital arena for political contention. While national figures, like the President, tend to dominate overarching narratives, it is those who represent state constituencies that adapt and translate these narratives into proximate, resonant appeals. This dynamic is especially pronounced given that local media tend to provide heightened coverage of national political developments as they relate to local representatives [68,69]. As Hunt and Rouse [70] note, state legislators, by virtue of their proximity to voters and grassroots networks, are uniquely positioned to shape local political climates and activate community-level mobilization. Meye [71] illustrates these dynamics in a recent study on anti-election protest mobilization following the 2020 U.S. presidential election. Specifically, when members Congress endorsed election denial narratives and amplified claims of fraud, protest activity increased within their congressional districts. In other words, local constituencies respond when their representatives legitimize and promote populist frames [72]. These findings resonate deeply with foundational scholarship on the role of local institutions in maintaining white supremacy throughout American history [73,74], and in how local political environments play integral roles in both the attitudes *and* behavior of community residents [75]. Even in the contemporary social media information environment and a context of the decline of local news [68,76], algorithms often promote geographically bounded content, such that elites are plausibly most likely to gain exposure amongst their constituents, on both legacy and new media [77].

However, at the same time, the influence of political elites is not strictly confined to their formal jurisdictions. Political messaging can diffuse across geographic boundaries, extending the reach of leaders beyond their immediate constituencies. Accordingly, by examining the interplay between elite endorsements, public attention, and mobilization behavior across both national and state levels, we can disentangle how these associations operate across different spatial scales.

### Core hypotheses

Integrating these theoretical and empirical observations leads us to four main hypotheses concerning the relationship between elite endorsements of white replacement conspiracies that are picked up within mainstream media outlets and their potential effects on (1) public attention and (2) mobilization or action. The first two hypotheses address these dynamics at the national level, and the third and fourth extend the tests to the subnational context of the state.

*Hypothesis 1:* The cumulation of public endorsements of white replacement conspiracy theories by politicians, that are reported by the media, will lead to increased national attention on immigration.
*Hypothesis 2:* The cumulation of public endorsements of white replacement conspiracy theories, that are reported by the media, will lead to increased levels of white supremacist activity in the United States.
*Hypothesis 3*: The cumulation of public endorsements of white replacement theory by state leaders, that are reported by the media, will lead to increased levels of attention toward immigration in their states.
*Hypothesis 4*: The cumulation of public endorsements of white replacement theory by state leaders, that are reported by the media, will lead to increased levels of white supremacist activity in their states.

## Analytic approach

This research integrates data from multiple sources. We begin by examining the data descriptively and assessing the patterns over time. We then test our hypotheses using different units of analysis and methodological approaches. First, we test hypotheses 1 and 2 using a national-level weekly time-series model, assessing how political endorsements of white replacement conspiracy theories that are reported by media might shape national attention and mobilize extremism. Second, we test hypotheses 3 and 4 using state fixed effects analysis on state-level, monthly panel data. We use monthly data to balance data sparsity with granularity at the more localized level. Together, these methods offer an expansive approach to understanding the potentially complex relationships between elite endorsements of replacement conspiracies, public attention, and extremist mobilization.

### Data

Because our central focus is on the consequences of political leaders publicly endorsing white replacement theories, we begin by explaining how we constructed this independent variable. All data collection and analyses complied with the terms and conditions for all sources of the data that were not originally collected by the authors.

#### Endorsements of white replacement conspiracies by public officials that gained media attention.

To measure endorsements of white replacement conspiracies by public officials that gained attention in mainstream media outlets, we created a new dataset that includes the endorsement date, endorser’s name, state representation (if applicable), and current office status. We define a white replacement endorsement as any public statement that (i) references immigration as a (ii) deliberate plot to (iii) replace the “true” people. For example, statements about an alleged invasion, even if blamed on the Democratic Party, are not considered an endorsement of white replacement unless they include an explicit narrative of *replacement*. Importantly, these criteria capture “replacement lite” (where the “true” population is Republican voters, implicitly white Christians), encompassing a range of endorsements that may appeal to white nationalists.

As emphasized above, we exclusively include endorsements reported by mainstream media platforms to capture only those statements that break through the noise of political competition and reach a broader public audience. By focusing on this subset, we capture the full universe of relevant cases within the specified time window. While it is likely that other political figures have endorsed white replacement theory, their statements have not garnered sufficient media exposure such as to plausibly direct public attention or mobilize white supremacist activity.

The original endorsement can be from social media, televised appearances or debates, news broadcasts, or any other public statement made to an audience. We included statements made by federal and state politicians holding office at the time, as well as by candidates leading up to an election. To identify these endorsements, we used case-insensitive Boolean searches on Google News and LexisNexis: (“Great Replacement” OR “White Genocide”) AND (“Representative” OR “Senator” OR “Governor” OR “Legislator” OR “Delegate”). We reviewed articles until no new cases emerged.

We identified 25 public endorsements, reported in the media, that were made by 16 individuals between January 2021 to December 2023, chronicling the original date for each statement. Because media reports of public statements remain available for viewing and sharing long after the original event, a central point of our theoretical reasoning, we measure the cumulation of endorsements (as opposed to only recording each statement during the period it was made) by calculating their cumulative frequency for each time period. For example, the above-mentioned endorsement of Great Replacement by Republican Presidential candidate Vivek Ramaswamy in a 2023 debate was reported by WIRED, The Atlantic, ABC News, The New Republic, and The Daily Beast, among others, and each story remains accessible well beyond December 2023. To test H1 and H2, the cumulative frequencies are measured each week. To test H3 and H4, when applicable, we link each political endorsement to the state the endorser represents, calculating the cumulative frequency on a state-month basis. This approach allows us to track both the emergence and persistence of white replacement endorsements by political elites across and within different contexts. A list of endorsements is included in Appendix A. Due to personally identifying information, we do not list the speaker’s name. A full dataset may be made available on request to the corresponding author.

#### White supremacist activity.

To measure white supremacist activity, we use data from the Anti-Defamation League’s (ADL) Hate, Extremism, Antisemitism and Terrorism Map (H.E.A.T.), an open-source database compiled from news reports, government documents, and extremist-related sources (e.g., the Telegram postings by extremist groups). While relying on open-source data likely misses less publicized events that are undocumented by the media, research centers, or the extremist groups themselves, these data are the most comprehensive source of white supremacist activity presently available.

These data include the date, city, ideology, group name (if known), type of activity (propaganda distribution or event), and a description of the incident. Most propaganda incidents are dated for the first of the month, indicating uncertainty about the exact distribution day. For example, on “October 1, 2022” in Midwest City, OK, the Patriot Front distributed propaganda that read “America first” and “For the nation, against the nation,” which could have been distributed any week in October. Due to this dating issue, we use propaganda data only for monthly analyses that test Hypotheses 3 and 4.

White supremacist events, such as mobilizations and protests, are assigned specific dates. For example, on August 13, 2022, in Nashua, NH, approximately “six individuals associated with the neo-Nazi Nationalist Social Club displayed a banner that read: ‘#SEND THEM ALL BACK. Defend New Hampshire.’” We exclude white supremacist murders as events as, though important to understand extremist activity, they are exceedingly rare and not fully aligned with our more general interest in mobilization. Note, results are however consistent when these are included in the counts of incidents. The data cover incidents from January 1, 2021, to December 31, 2023, totaling 609 events and 19,175 incidents of propaganda. This period is crucial due to the impact of the January 6^th^ insurrection on extremist group behavior (Armed Conflict Location and Event Data 2022, 2023). The count of events is aggregated to the weekly-level to test H1 and H2 and to the monthly-level for tests of H3 and H4.

#### Public attention on immigration and immigration-related conspiracies.

Public attention towards immigration and white/replacement conspiracies are calculated using Google Trends data from U.S. searches. These data, anonymized and categorized by Google, are drawn from a random sample of daily searches and aggregated to the requested spatial-temporal units, and released as the number of searches made on specific topics relative to all other Google searches. This search-based measure is particularly advantageous as it provides a population-centric and agency-based metric of attention (i.e., the public), directly aligned with our theory. Additionally, capturing relative public attention makes the analysis less vulnerable to biases due to circumstances that would influence the volume of Google searches (e.g.,., internet coverage, population growth, access to technology).

A key advantage of Google Trends is the ability to capture topics over simple keyword-based searches. Topics are identified via the Google Knowledge Graph, which clusters semantically related queries rather than relying on exact string matches. This offers several benefits. First, topics capture searches across multiple languages and phrasings, meaning that a query entered in Spanish, Mandarin, or any other language is attributed to the same underlying topic. This is particularly important in the U.S. context, where a substantial share of the population searches in languages other than English. Second, topic-based aggregation better approximates user intent: a Boolean approach matching on a specific term inevitably conflates homonyms and misses synonyms, whereas the Knowledge Graph disambiguates meaning. For instance, while a keyword search for “immigration” captures queries like “immigration reform bill” or “immigration at the border,” it misses Spanish-language searches like “trámites de migración” or related English queries like “illegal aliens.” A topic-based approach correctly groups these together under the broader concept of immigration. Third, topics subsume a far larger and more diverse set of query strings than any researcher-specified keyword list could anticipate, capturing colloquial expressions and misspellings, that a fixed Boolean filter would most likely miss. The result is a measure that more closely reflects the breadth of public attention to a concept as people actually express it.

Recent work has raised concerns about the reliability of Google Trends data. Cebrian and Domenech [78], in particular, show that repeated queries for the same search term at different points in time can return different trend values, suggesting instability in Google’s underlying sampling procedure. We take this concern seriously. To assess whether it affects our analysis, we compare immigration-topic data retrieved during the initial drafting of this paper (early 2025) with data retrieved during revisions (May 2026). While the two series differ slightly, they are very highly correlated (r = 0.98), indicating that any changes to Google’s sampling algorithm over this period had negligible effects on the relative trends we exploit. Moreover, our main results are robust to using either iteration of the data, offering further reassurance that our findings (discussed later) are not an artifact of a particular data pull.

To test H1 and H3, which anticipate that the accumulation of public endorsements of white replacement conspiracy theories that were reported in media will lead to increased attention on immigration, we obtain the weekly dependent variable of *immigration searches* at both the national and state geographic unit. We were also interested in looking at more explicit searches for “white replacement.” Unfortunately, these searches are much rarer overall and thus the sampling strategy is unable to reliably estimate the relative attention level, especially at the state level. We therefore proceed with general attention on immigration.

#### Control variables.

We also include a set of control variables that may confound the relationship between elite endorsements, attention, and mobilization. As we detail in the next subsection, our modeling strategy accounts serial dependencies (at the national-time series level) and for all time-invariant state characteristics or cross-state trends (at the state-panel level). However, confounders can still exist via relevant phenomenon that move differentially across states over time.

First, then, we include a control variable that indicates whether the week or month falls within the general election season for 2022. This variable is coded as 1 in the weekly time series data beginning in the first week of September, which is after the last state primary. It turns back to zero after the first week of November. For the state-level models, this variable is coded as 1 beginning in the month following each state primary. Louisiana has a “jungle primary” in which all candidates run against each other on election day. We set the start date of their election season as the median month of last primary for all other states: June 2022. It turns back to zero in December 2022 for all states. Georgia and Louisiana both had run-offs in the 2022 midterms. We extend their end date to include December 2022.

One major source of hostility toward immigrant populations has been the framing of immigrants as violent criminals [2], despite the persistent finding that homicide rates by immigrants and Latinos is lower than for other groups. We therefore assess whether the number of homicides perpetrated by Hispanic offenders might confound the hypothesized relationships. Hispanic homicide data are taken from the Supplementary Homicide Reports, which is part of the Uniform Crime Reports program and collected by the F.B.I. These data are recorded at the incident level, but participation in the program is voluntary. Furthermore, offender data are only available when the offender is known, which depends on whether the case is solved, and the record is updated prior to submitting it to the UCR program. While these issues do raise caution on the validity of this variable, its missingness is unlikely to be related to our outcome variables. Separate analysis instead using the total homicide count as a control produce the same finding as Hispanic homicide, relieving some concern. Finally, only the month and year are recorded in the data, which means that the weekly time-series analysis will have the same values for weeks that fall within the same month. This deflates the standard errors, leading to possible false significance for that specific variable. Given that we are only using homicide as a control variable, this is not a concern.

### Methodology

We test the hypothesized relationships at both the national (time series) and state (longitudinal, panel analysis) levels.

#### National-level analysis: time series models.

To test hypotheses 1 and 2, we run weekly time series analysis for the nation using Google Trend searches on immigration as the dependent variables for the first hypothesis and white supremacist events as the dependent variable for the second. White supremacist events are transformed using their square root to better approximate a normal distribution. To account for dependencies across the error terms, we model the data using Auto Regressive Moving Average with eXogeneous variables (ARMAX) model. The ARMAX component captures two types of error dependencies while simultaneously estimating the effects of key independent variables, treating them as exogenous influences with their own temporal dynamics [79]. Nonstationary dependent variables are differenced as part of the estimation process. Model parameters are estimated using the Hyndman-Khandakar algorithm [80] through a combination of unit root tests, and minimization of the AIC and MLE. We also include a measure of seasonal strength, where identified as relevant, from an STL decomposition. To test the first hypothesis, we run the model shown in equation 1.


$$\begin{array}{c} Attentiont\;=\text{ f}({Endorsements}_{t},\;{White\;Supremacist\;Eventst}_{t},\;{Election}_{t},\;{Homicide}_{t}) \end{array}$$ (1)

Here, *Attention* refers to immigration searches for week *t* on Google and the other variables are measured during the same week *t*. We include the count of white supremacist events as a control, as this may well also influence public attention. As mentioned above, we also control for whether the weeks are within an election season. To test the second hypothesis, we run the ARMAX model shown in equation 2 and add controls for general levels of public attention on the dimensions of immigration and for whether the week was during the election season.


$$\begin{array}{c} {White\;Supremacist\;Events}_{t}=\text{ f}({Endorsements}_{t},\;{Immigration\;Att.}_{t},\;{Election}_{t},\;{Homicide}_{t}) \end{array}$$ (2)

#### Fixed-effects models for panel data.

To test hypotheses 3 and 4, we use monthly level state fixed effects regression models. These models account for all heterogeneity that is specific to each state and remains consistent across the entire study period, which captures time stable heterogeneity within each state. We also control for month, which captures all seasonal patterns that are the same across all states. This allows us to estimate the effects of changes on changes for each state, while controlling for any regular monthly trends across all states (Greene 2019). To test the third hypothesis, we transform the Google Trends immigration searches using its square root to better approximate normality (equation 3). For the fourth hypothesis, we use negative binomial fixed-effects regression models because the number of both white supremacist events and propaganda distributions are relatively rare across state-months (see equation 4) and show significant (p < 0.05 and p < 0.001, respectively) evidence of over-dispersion [81]. We include state-clustered standard errors in all fixed-effect models. We note there may be some spatial spillover between states, especially for statements that gain national attention. However, as our outcome is constrained to activity within states, this would bias our estimates toward zero. Thus, our state-level findings likely reflect conservative estimates.


$$\begin{array}{c} {Immigration\;Att}_{it}\;=\text{ f}({Endorsements}_{it},\;{White\;Supremacist\;Events}_{it},\;{Election}_{it},\;{Homicide}_{it},\;{Month}_{it}) \end{array}$$ (3)

$$\begin{array}{c} {Extremist\;Activity}_{it}=\text{ f}({Endorsements}_{it},\;{Immigration\;Att.}_{it},\;{Election}_{it},\;{Homicide}_{it},\;{Month}_{it}) \end{array}$$ (4)

#### Sensitivity analyses.

To assess the robustness of our results to alternative modeling specifications we run the following additional analyses and discuss the results in text where relevant. The full tabular output of the alternative analytical methods is available in an online appendix. First, both the time series and fixed effects models are modeled with cumulative number of endorsements lagged to impose temporal ordering. Second, models where the outcome variable is transformed to better approximate normality are re-run using an ordered quantile normalization (ORQ) rank transformation that forces normality [82]. Though interpretation is opaquer, these analyses ensure that any significant results are independent of the transformation procedure. Finally, though our focus is on the effects of cumulative endorsements, which is best accommodated by the fixed effects approach to testing H3 and H4, we recognize that this raises concerns of bias due to staggered treatment effects [83]. Though, we note our cumulative model is not a “treatment” in classical terms (i.e., we have multiple), we test the robustness of our findings by setting the first endorsement as the main “treatment” and applying the Sun and Abraham method, which estimates a series of treated cohort × time to treatment dummies which are later aggregated to obtain the average treatment for the treated (ATT) for the post treatment period. The results of these alternative approaches are generally consistent with the main findings, albeit with some important nuance regarding the immigration attention variable, which we discuss accordingly.

## Results

Table 1 lists the mean, standard deviation, range, and percentage of the observations with zero value for both analyses. Turning first to our key independent variable, we see from the range that we begin both analysis with zero endorsements—as we excluded endorsements that were made prior to 2021—and end with 25. Between 2021 and 2023, there were 609 white supremacist events. All but 10% of the weeks had at least one event, while at least one week had 15 events, averaging 3.8 white supremacy events per week. When spread across the states, the average state-month was 0.34, or one event every 3 months, such that most state-months had no white supremacy events. In contrast, the data record 19,109 white supremacy propaganda events in these years. Only 15.6% of the state-months had no recorded distribution of propaganda. On average, each state-month had just over 10 propaganda events. Because immigration searches are relative measures to total search volume, we turn to their patterns over time rather than interpreting their summary statistics.

**Table 1:** Summary statistics of key dependent and independent variables.

	Week (n = 157)				State-Month (n = 1,800)			
	Mean	St. Dev.	Range	% Zero	Mean	St. Dev.	Range	% Zero
Cumulative Endorsements	15.82	8.37	0, 25	8.28	0.32	0.89	0, 5	83.78
White Supremacy Events	3.88	2.88	0, 15	10.19	0.34	0.83	0, 13	77.72
White Supremacy Propaganda	--	--	--	--	10.62	14.03	0, 128	15.56
Immigration Searches	46.60	9.98	26, 98	0.00	15.87	4.98	0, 56	0.06
Election Season	0.14	0.35	0, 1	86.00	0.14	0.35	0, 1	86.11
Hispanic Offender Homicides	93.46	19.58	59, 136	0.00	1.95	4.75	0, 40	51.34

**Fig 1:**
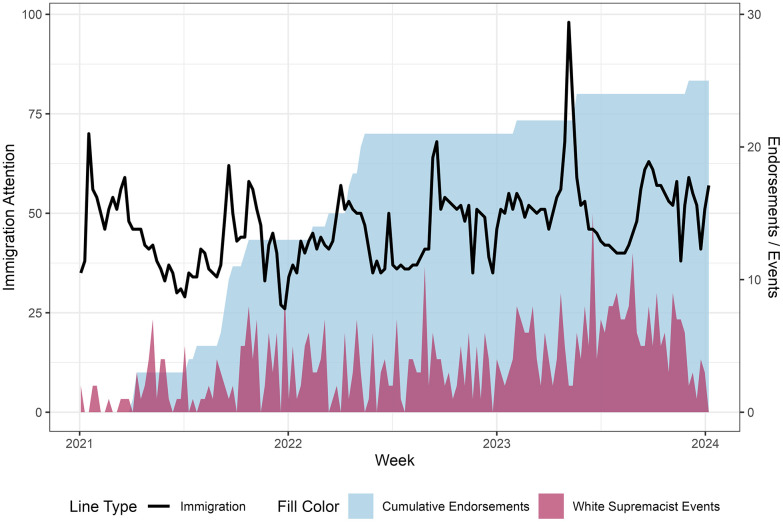
Weekly measures of attention, cumulative endorsements, and white supremacist activity, 2021–2023.

Fig 1 presents the weekly trends of the three key variables used in the time series models, from January 2021 to December 2023. Endorsements and White Supremacy Events are presented as unstacked area plots, which are measured on the right axis. The Google Trends search for immigration is shown as a line and measured on the left axis. We see from the gray area representing the cumulative endorsements that there were quick increases just shy of 40 weeks into 2021 in September, and again around 20 weeks in 2022 in April. When we examine the white supremacist activity, we see that it was more sporadic in early 2021 but then began to pick up in 2022 and 2023. Note that this figure excludes White Supremacy propaganda, as it was only measured at the monthly level. For attention on immigration, we see some fluctuation, but with a clear peak in the middle of 2023 (May). While we cannot exactly identify why this peak exists, we note it coincides with not only the peak in white supremacist events (albeit that comes slightly later) but also several high-profile policy events. In particular, the expiration of Title 42 in May 2023 under the Biden administration, a pandemic era policy that allowed for the rapid expulsion of migrants at the U.S.-Mexico border and the end of which saw a rapid increase in immigration levels.

### Tests of hypotheses 1 and 2

Turning to the hypothesis tests, we first examine the results for the weekly ARMAX models that use public attention toward immigration as the dependent variables to test Hypothesis 1. Table 2 presents the findings of this model in the first column. The table lists the coefficient estimates and their standard errors in parentheses with notations of significance for two-tailed tests. Continuous control variables (i.e., other than endorsements) are scaled and centered. The error structure needed to remove dependencies in the error terms are listed below the estimates, and the AICs and BICs are listed at the bottom. Ljung-Box tests confirm that autocorrelation is removed from the ARMAX models. We see the first hypothesis is unsupported at the conventional p < 0.05 level of significance. However, a closer look reveals some evidence that attention toward immigration might increase with the cumulation of public officials endorsing white replacement theories, as the p-value only just falls above the typical threshold (p = 0.07).

**Table 2:** Coefficient estimates and standard errors for ARMAX models predicting attention on immigration and white supremacist events.

	Public Attention	Extremist Activity
	***Immigration***	***Events (sqrt)***
C. Endorsements	0.419 (0.234)	0.058*** (0.01)
Immigration Attention	–	−0.012 (0.065)
Election Season	1.25 (4.74)	−0.256 (0.255)
Hisp. Homicide	−0.938 (1.459)	−0.09 (0.088)
White Supr. Events	0.007 (0.188)	–
Error Structure	AR1	AR0
AIC	1062.11	365.33
BIC	1083.51	383.67

* p < 0.05, ** p < 0.01, *** p < 0.001, all tests are two-tailed.

The test result for Hypothesis 2 is found in the second column of Table 2, which presents a positive and significant association between cumulative endorsements of white replacement conspiracies and white supremacist activity. This result supports Hypothesis 2, suggesting that public endorsements of white replacement conspiracies that achieved media attention contribute to an increase in white supremacist extremist activities. These findings remain substantively similar when the models are run with 1) a lagged independent variable for cumulative endorsements and 2) ORQ transformed dependent variables (when applicable) to force normality through rank transformations. In fact, we see the immigration association move below p < 0.05 to reach conventional levels of significance in both of the alternative specifications.

### Tests of hypothesis 3 and 4

We now turn to the tests for Hypotheses 3 and 4 that as the cumulative number of public endorsements of white replacement conspiracies by representatives of the *state* increases, so will public attention toward immigration (H3) and white supremacy activity (H4). Table 3 presents the coefficients and standard errors of the state fixed-effects models. Here we see that both hypotheses are supported as all three coefficient estimates for cumulative endorsement are positively and significantly associated with the outcomes. In support of Hypothesis 3, with each additional endorsement of a white replacement conspiracy the square root of immigration searches in the endorser’s state increases. Recall that this coefficient was only marginal when measuring changes in the entire country when testing Hypothesis 1.

**Table 3:** Coefficient estimates and standard errors for state fixed-effects models predicting public attention on immigration, white supremacy events, and propaganda.

	Public Attention	Extremist Activity	
	***Immigration (Sqrt)***	***Events***	***Propaganda***
C. Endorsements	0.0522* (0.0201)	0.4103*** (0.0708)	0.1819** (0.0673)
Immigration Attention	–	0.0121 (0.0816)	0.1876*** (0.0469)
Election Season	−0.0545** (0.0181)	−0.4475* (0.2060)	− 0.1339 (0.0985)
Hisp. Homicide	−0.0020 (0.0028)	0.0661*** (0.0194)	0.0030 (0.0188)
White Supr. Events	0.0121 (0.0079)	–	–
White Supr. Prop.	0.0017 (0.0075)	–	–
Model Type	OLS	Negative Binomial	Negative Binomial
Obs.	1716	1543	1685
Adj. Pseudo R^2^	–	0.173	0.110
Adj. R^2^	0.797	–	–

* p < 0.05, ** p < 0.01, *** p < 0.001, all tests are two-tailed. State-clustered standard errors in parentheses. All models include state fixed-effects and month dummies.

These models produce comparable results when estimated with a lagged predictor for endorsements. We also estimate the average treatment effect on the treated (ATT) using the Sun and Abraham (2021) estimator for staggered difference-in-differences designs, where the first endorsement in a state constitutes treatment onset. This approach yields a positive association between a state’s first endorsement and subsequent levels of events and propaganda. The relationship with immigration attention is, however, no longer significant at the standard p < 0.05 level (though, we note that it is still marginally significant (p = 0.066). The difference-in-differences specification is a distinct modeling framework with a separate interpretation: notably, it considers only the first endorsement and therefore sets aside the cumulative dynamics that are central to our main analysis (and reduces variation in our independent variable). Nonetheless, the consistency of the events and propaganda findings across these alternative specifications strengthens our confidence that the association between endorsements and public behavior is not an artifact of any particular modeling choice.

Turning to the reduced significance of the immigration attention model in the difference-in-differences framework, we note that attention is likely governed by a more dynamic process than events. Legislators may not only shape public attention through their endorsements but also strategically increase their immigration rhetoric as public interest in the topic rises. To probe this possibility, we estimated additional models predicting cumulative endorsements as a function of lagged immigration attention. In the national time-series specification, we find no evidence of reverse causality. At the state level, however, we do find a significant positive association between immigration attention in a given month and cumulative endorsements in the following month (p < 0.01). This finding prods some interpretive caution. While our main models identify an association between endorsements and immigration attention, consistent with our theory, the state-level evidence suggests the relationship may be bidirectional or otherwise more co-constitutive (i.e., with endorsements and public attention mutually reinforcing one another) rather than strictly unidirectional.

## Discussion

This paper examined whether elite endorsements of white replacement conspiracy theories may shift public attention and mobilize white supremacy in the United States. Our findings are consistent with theoretical expectations that they do. Specifically, we assessed whether the cumulation of endorsements of white replacement conspiracies by political leaders is associated with (1) public attention on immigration-related topics and (2) white supremacist activity in the United States. Recognizing the complex and intersecting dynamics between political narratives, cultural climates, and individual behavior, we compiled a novel dataset comprising explicit endorsements of white replacement narratives (that gained media attention) by federal and state legislators and candidates for office, Google trends data, and information on white supremacist events and propaganda. Effects were estimated for both the entire nation through time series analysis and for the fifty states using fixed effects panel models.

Our first and third hypotheses claimed that as endorsements of demographic conspiracies by politicians (that are reported by media) accumulate, people will turn their attention to immigration. We found marginal evidence for a relative rise in immigration-related Google searches at the national level. This suggests that these endorsements may shift attention towards immigration, as people seek additional information on what is happening at U.S. borders, and beyond. Notably, this relationship was especially pronounced in the state-level models, suggesting that such endorsements may be particularly effective for shaping the attention of a politician’s own constituents. In this regard, our analyses concur with recent studies highlighting the importance of local elites in shaping local political attitudes and behaviors [71].

Our second and fourth hypotheses consider the role that political endorsements of white replacement conspiracy theories play in communicating to white supremacists that elites are aligned with their extreme beliefs. Both explicit endorsements of white replacement conspiracies and dog whistles, like the “Replacement Lite,” signal that extreme ideas have gained legitimate grounding within the political mainstream. This, in turn, may *embolden* white supremacist actors to more openly engage in anti-immigration efforts [16] or, equally plausible, motivate increased action to seize on the *discursive opportunities* of their views being amplified by mainstream political elites and media outlets [17,66]. Our findings support these hypotheses at both levels.

When political leaders accuse Democrats of allowing immigrants to illegally enter the country to vote against Republicans, white supremacist groups appear to become more active across the nation. Indeed, states with at least one leader who endorsed white replacement theories had, on average, more white supremacist events (0.59 vs. 0.25, p < 0.001) and more propaganda (15.93 vs. 9.29, p < 0.001). Furthermore, white supremacist events (0.67 vs. 0.71, p < 0.05) and propaganda (17.44 vs. 10.46, p < 0.001) were more frequent in the endorsement states after the first endorsement than before. This observation speaks directly to the role of elite rhetoric in shaping hostile political mobilization against minority racial and ethnic groups [63,66], but also much more broadly to the discursive and cultural components of social movement mobilization [18–20,84]. These results should raise further concerns about the mainstreaming of extreme positions seen in the United States in recent years (facilitated by political rhetoric emanating from both national and local government representatives).

Though important findings, we must qualify them with some limitations. First, though each public endorsement gained high public exposure by being reported on by a mainstream media outlet, we were unable to measure differences in levels of exposure. Once a story is released, it is often picked up by other (potentially local) outlets and spread by individuals, including in-person communication, in unmeasurable ways. Furthermore, this key independent variable is treated as a step function that rises only during the week or month of each endorsement and remains flat in-between. However, it is likely that between each endorsement, exposure increases exponentially with a form of contagion, making our measure a somewhat crude proxy for true societal exposure. As such, we expect our findings to in fact underestimate any real effect as movement is likely more continuous than our measure shows.

A second limitation is that Google Trends data only provides a measure for searches for specific topics relative to all other searches on that search engine. While limiting our sample to only those searches made from within the United States or within each state does mean this measure is able to capture shifts in public attention, these shifts reflect proportionate changes rather than absolute shifts. Identifying ways to measure whether these shifts reflect a genuine increase in the volume of searches would require data that are currently unavailable. Having said that, if we are willing to believe that public attention remains a constant commodity, then relative attention would approximate absolute attention. This means our findings suggest that some people turn their limited attention, within an attention economy, toward immigration rather than what they would otherwise focus upon. A related concern with the immigration attention variable is reverse causality, in which endorsements may be strategically made *in response to* rising attention on immigration. Our supplementary models with lagged predictors, indeed, suggest both directions are plausible. Though not detracting from our main theory, the bidirectional relationship between endorsements and immigration attention is a potentially compelling topic for future research.

Despite these limitations, our findings illuminate critical dynamics at the intersection of demographic change, political rhetoric, and societal responses. Specifically, we have shown that as political endorsements of white replacement theories gain traction and media visibility, public attention to immigration intensifies and white supremacist activity escalates. These findings highlight the powerful role political elites may play in shaping public discourse *and* mobilizing extremism. This study therefore contributes to a growing body of research examining the link between hostile political/public discourse and hostile actions [16,22,23], and the “political economy of hatred” more broadly [23,85], with a particular focus on narratives surrounding demographic change *and* applied to the case of protest (i.e., not just violence). In doing so, we emphasize the urgent need to hold political elites accountable for their hostile rhetoric. Moreover, the pronounced association between elite discourse and public attention at the state level highlights the importance of examining how extremist ideas, such as white replacement conspiracies, circulate within subnational political institutions [71,86,87].

We wish to note, in closing, that the data for this study measured activity in the United States when a Democrat was in control of the White House. Future research should unpack the extent to which similar patterns exist when replacement conspiracies are endorsed by active members of the controlling party. Are such statements now more frequently invoked? Are they, in fact, less impactful as numerous similar statements saturate the discursive environment? Future research should also explore whether these patterns hold across different types of white replacement conspiracies, such as those centered on birth rates [88], and the potentially differential influence of various types of political actors and mediums of communication. Additionally, further work is needed to assess how political endorsements of conspiracy narratives disseminate across both mainstream and alternative online platforms, alongside how exactly these narratives may translate into real-world mobilization [64]. This point, in particular, would benefit from a mixed-method approach that incorporates qualitative analyses to more exactly unpack the ongoing mechanisms for any aggregate patterning. We urge scholars to more fully examine the potential implications of political rhetoric surrounding demographic change.

## Supporting information

S1 File The supplementary materials include a list of each endorsements’ content (Appendix A) and the results of supplementary statistical models (Appendix B). (DOCX)
