# Supplementary material for: Endorsing narratives of white replacement: Elite discourse, public attention, and extremist mobilization
Source: PLoS One. 2026 Aug 5;21(8):e0353353. doi: 10.1371/journal.pone.0353353 (PMC13440818; doi:10.1371/journal.pone.0353353)
Supplement: Supplementary file 1 [file pone.0353353.s001.docx]

**Appendix A.** Endorsements of Demographic Replacement Conspiracies.

**Statement**

The truth is, they want borders wide open… It helped Democrats take over the entire state of California. … We have to take our country back.

For many Americans, what seems to be happening or what they believe right now is happening is what appears to them is we’re replacing national-born American — native-born Americans to permanently transform the landscape of this very nation.

She [Kamala] is fully aware of this, and yet this administration wants complete open borders. And you have to ask yourself why? Is it really they want to remake the demographics of America to… stay in power forever? Is that what's happening here?

We are being replaced and invaded.

[After FOX Business host Dagen McDowell stated that immigration “increases the voting base for the Democrats”] that’s right, we are not conspiracy theorists but that is the only thing that makes sense here… they want to turn these people into voters.

They want to replace the American electorate with a Third World electorate that will be on welfare.

@TuckerCarlson is CORRECT about Replacement Theory as he explains what is happening to America. The ADL [Anti-Defamation League] is a racist organization.

They want to grant amnesty and a path to let in citizenship to 8 million illegal aliens. Yes, there is definitely a replacement theory that’s going on right now. We are killing American jobs and bringing in illegal aliens from all over the word world to replace them if Americans will not comply with the tyrannical orders that are coming down from the White House. This Democrat bill puts America dead last, and sadly, this is what Americans have come to expect from [the Biden administration].

The revolution has begun. A silent revolution by the Democrat Party and [President] Joe Biden to take over the country. When I say a revolution has begun, they are allowing this year probably 2 million—that is who we apprehended, maybe another million—into this country… you’re talking about millions and millions and millions of new voters, and they will thank the Democrats and Biden for bringing them here. Who do you think they are going to vote for?

Radical Democrats are planning their most aggressive move yet: a PERMANENT ELECTION INSURRECTION. Their plan to grant amnesty to 11 MILLION illegal immigrants will overthrow our current electorate and create a permanent liberal majority in Washington.

[There’s an Arabic term that says] hey we don't need to beat them with weapons, we'll beat them with our bellies, meaning if we move into these countries and just through birth rates have a lot more kids than they do we will use their constitution and use their laws against them…

Joe Biden, Nancy Pelosi, Chuck Schumer,

with Obama pulling strings behind the scenes and Soros funding it, that is exactly what they want to do in the United States if they flood the southern border with illegals.

This is funded by Soros ... and the Obama cabal.... they are intentionally flooding the border... this is about changing the face of America... they are trying to change our culture, our demographics, and our electorate.

What the left really wants to do is change the demographics of this country. They want to consolidate power so they can never lose another election … we don’t need to import more than a million people every year to replace Americans who were born here.

Biden has looked at his approval ratings and realizes that the only way to win in ‘24 is to replace the citizens of his own country with illegal foreigners. The invasion he’s allowing to happen at the border is about power for democrats and nothing more.

Democrat politicians who have decided that they can’t win re-election in 2022 unless they bring in a large number of new voters to replace the voters that are already here.

There are somewhere between 200 to 250,000 immigrants a month… that’s going to be 10 million to 15 million people… you are talking about a demographic shift in our country that means we never win, the Republicans never win a national election in this country ever again.

Obviously, the Democrats, they hope to just change the demographics of our country… they hope to import an entirely new electorate. Then they call you a racist and a bigot.

They are fundamentally trying to change this country through their illegal immigration.

HERE’S THE ANSWER for all who have asked why in the world the Democrats would intentionally open our borders: Late last night I got them to admit the goal is to TURN ILLEGALS INTO VOTERS!

Democrats desperately want wide open borders and mass amnesty for illegals allowing them to vote.

The Democrats want open borders so they can bring in and amnesty tens of millions of illegal aliens, that’s their electoral strategy. Not on my watch.

Why would Biden do this? [let in more immigrants]... more control, even changing our culture.

The Biden administration has done this intentionally… For what reason? Everybody asks me all the time. I think that, ultimately, they hope to turn all these illegals into voters for their side.

The millions of illegal aliens pouring into America are a result of a cynical political decision by Biden, Harris, and Congressional Democrats. They believe each illegal alien is a potential Democrat voter.

The great replacement theory is not some grand right-wing conspiracy, but a basic statement of the Democratic party's platform.

**Appendix B:** Coefficient, Standard Errors, and Significance Level of Alternative Specifications for Cumulative Endorsements.

| **Level** | **Outcome** | **Alternative Specification** | **Original Model** | **Alternative Model** |
| --- | --- | --- | --- | --- |
| National | Attention | Lagged Endorsement | 0.419  (0.234) | 0.484*  (0.192) |
| National | Events | Lagged Endorsement | 0.058***  (0.010) | 0.058***  (0.011) |
| National | Attention | ORQ Outcome | 0.419  (0.234) | 0.053**  (0.020) |
| National | Events | ORQ Outcome | 0.058***  (0.01) | 0.064***  (0.011) |
| State | Attention | Lagged Endorsement | 0.052*  (0.020) | 0.062**  (0.023) |
| State | Events | Lagged Endorsement | 0.410***  (0.071) | 0.409***  (0.052) |
| State | Propaganda | Lagged Endorsement | 0.182**  (0.067) | 0.186**  (0.063) |
| State | Attention | Sun and Abraham (ATT) | 0.052*  (0.020) | 0.136  (0.072) |
| State | Events | Sun and Abraham (ATT) | 0.410***  (0.071) | 2.134***  (0.208) |
| State | Propaganda | Sun and Abraham (ATT) | 0.182**  (0.067) | 0.448*  (0.210) |
